# BMAL1 controls the diurnal rhythm and set point for electrical seizure threshold in mice

**DOI:** 10.3389/fnsys.2014.00121

**Published:** 2014-06-26

**Authors:** Jason R. Gerstner, George G. Smith, Olivia Lenz, Isaac J. Perron, Russell J. Buono, Thomas N. Ferraro

**Affiliations:** ^1^Department of Neuroscience, University of PennsylvaniaPhiladelphia, PA, USA; ^2^Center for Sleep and Circadian Neurobiology, University of PennsylvaniaPhiladelphia, PA, USA; ^3^Department of Psychiatry, University of PennsylvaniaPhiladelphia, PA, USA; ^4^Research Service, Department of Veterans Affairs Medical CenterCoatesville, PA, USA; ^5^Department of Biomedical Sciences, Cooper Medical School of Rowan UniversityCamden, NJ, USA

**Keywords:** ARNTL transcription factors, circadian rhythm, CLOCK proteins, epilepsy, epileptogenesis, MOP3

## Abstract

The epilepsies are a heterogeneous group of neurological diseases defined by the occurrence of unprovoked seizures which, in many cases, are correlated with diurnal rhythms. In order to gain insight into the biological mechanisms controlling this phenomenon, we characterized time-of-day effects on electrical seizure threshold in mice. Male C57BL/6J wild-type mice were maintained on a 14/10 h light/dark cycle, from birth until 6 weeks of age for seizure testing. Seizure thresholds were measured using a step-wise paradigm involving a single daily electrical stimulus. Results showed that the current required to elicit both generalized and maximal seizures was significantly higher in mice tested during the dark phase of the diurnal cycle compared to mice tested during the light phase. This rhythm was absent in BMAL1 knockout (KO) mice. BMAL1 KO also exhibited significantly reduced seizure thresholds at all times tested, compared to C57BL/6J mice. Results document a significant influence of time-of-day on electrical seizure threshold in mice and suggest that this effect is under the control of genes that are known to regulate circadian behaviors. Furthermore, low seizure thresholds in BMAL1 KO mice suggest that BMAL1 itself is directly involved in controlling neuronal excitability.

## Introduction

Time-of-day factors such as sleep and circadian rhythms are well known to influence expression of seizures (Matos et al., 2010a; Cho, 2012). Studies in humans have shown an association between seizures and alterations in sleep-wake states (Bazil and Walczak, 1997; Crespel et al., 2000; Herman et al., 2001; Dinner, 2002). In patients with common forms of idiopathic generalized epilepsy, tonic, and tonic-clonic seizures are more frequently seen in sleep, whereas all other generalized semiologic seizure types including clonic, myoclonic, absence and atonic occur more frequently out of wakefulness (Zarowski et al., 2011), suggesting circadian rhythms control distinct mechanisms of neuronal hyperexcitability that may result in seizures. Sleep is also associated with twice as many interictal discharges in patients with idiopathic generalized epilepsy, especially during the period of nonrapid eye movement sleep (Seneviratne et al., 2012). In patients with lesional epilepsy characterized by seizures originating from the frontal lobe, nearly three quarters of their seizures occur during sleep (Kaleyias et al., 2011). Furthermore, sleep not only increases the frequency of epileptiform abnormalities, but also alters electrographic seizure morphology and distribution (Sanchez Fernandez et al., 2013). Seizures can also influence changes in sleep. Epileptic patients demonstrate multiple sleep abnormalities, including increased sleep latency, fragmented sleep, increased awakenings and stage shifts, and an increase in stages 1 and 2 of nonrapid eye movement sleep (Foldvary-Schaefer and Grigg-Damberger, 2009).

Among many forms of human epilepsy, several associations between seizure expression and time-of-day are especially well documented. Included are benign Rolandic epilepsy of childhood, in which seizures occur most prominently during sleep (Beaussart, 1972), Autosomal Dominant Nocturnal Frontal Lobe Epilepsy (ADNFLE) in which seizures are most prominent following transition from wakefulness to sleep (Scheffer et al., 1994, 1995) and juvenile myoclonic epilepsy when seizures occur mostly in the transition from sleep to wakefulness (Gigli et al., 1992). In patients with non-lesional temporal lobe epilepsy (TLE), the highest frequency of seizures was observed to occur approximately in the middle of the wake period, peaking at 1500 h (Quigg et al., 1998). This is consistent with results from two other studies, one in which patients with TLE exhibited a peak in seizure frequency in the afternoon between 1500–1900 h (Pavlova et al., 2004) and another in patients with focal epilepsies experienced significantly greater numbers of seizures between 1100–1700 h (Hofstra et al., 2011). Evidence for bimodal increase in seizure frequency in TLE has also been reported with peaks in the morning and in the late afternoon (Durazzo et al., 2008; Karafin et al., 2010). Therefore, understanding the way sleep or circadian mechanisms play a role in time-of-day regulation of seizure occurrence has important clinical applications.

Associations between sleep and epilepsy are also observed in animal models (van Luijtelaar and Bikbaev, 2007; Matos et al., 2010a,b; Yi et al., 2012). Mice with targeted deletion of genes involved in diurnal rhythms have been particularly instructive. Spontaneous electrographic seizures were documented in mice lacking Pten, a tumor suppressor gene that also significantly affects free-running rhythm (Ogawa et al., 2007). Deletion of three distinct PAR bZip transcription factors, proteins that accumulate with strong diurnal rhythms in critical brain areas such as the suprachiasmatic nucleus of the hypothalamus, causes spontaneous seizures in mice and is associated with reduced threshold to audiogenic seizures (Gachon et al., 2004). Interestingly, interictal abnormalities, and occurrence of spontaneous behavioral seizures in triple knockout PAR bZip mice followed a circadian trend that paralleled the distribution of sleep. These data suggest that molecular mechanisms related to the circadian clock likely play a key role in establishment of seizure susceptibility.

BMAL1 (also known as Arntl or Mop3) is a basic helix-loop-helix-PAS domain containing transcription factor and a core component of the circadian clock (Ko and Takahashi, 2006). BMAL1 gene expression is necessary for the establishment of circadian rhythms under free-running conditions (Bunger et al., 2000). BMAL1 KO mice have a significant disruption of the normal diurnal distribution of NREM and REM sleep over the light-dark cycle (Laposky et al., 2005), suggesting that BMAL1 is necessary for time-of-day dependent behavior even in the presence of a zeitgeber. BMAL1 expression has also been shown to be affected by sleep loss (Maret et al., 2007), and can influence the homeostatic response to sleep deprivation (Laposky et al., 2005) and certain aspects of memory formation (Kondratova et al., 2010). In addition to sleep and memory, core molecular clock mechanisms influence synaptic plasticity, further evidence for a neurophysiological role outside of the establishment of circadian locomotor rhythms (Gerstner and Yin, 2010). In this study, we hypothesized that seizure threshold has a time-of-day dependent component regulated by BMAL1. We observed a strong diurnal regulation of both generalized and maximal seizure threshold in WT mice. This effect was blocked in the BMAL1 KO. We also observed that seizure endpoints are largely influenced by the light-dark cycle, and that in addition to the control of diurnal seizure susceptibility, BMAL1 is also necessary for baseline seizure threshold. Further, BMAL1 KO mice display reduced seizure endpoints, suggesting clock factors such as BMAL1 affect not only the temporal patterning of seizures, but also overall neural excitability.

## Methods

### Animals

All studies were approved by the Institutional Animal Care and Use Committees at the University of Pennsylvania and VAMC Coatesville. Experiments involved C57BL/6J wild type mice (The Jackson Laboratory, Bar Harbor, ME) and co-isogenic Bmal knockout (KO) mice (from G. FitzGerald). Mice used in these studies were bred in-house at the VAMC Coatesville vivarium. Bmal KO mice were maintained as a homozygous strain. Litters were weaned between 21–28 days and pups were group housed by sex until the age of 6 weeks when they were entered into the study. Mice were maintained on a 14/10 h light dark cycle with free access to food and water at all times.

### Zeitgeber entrainment

At the age of 6 weeks, mice were randomly assigned into one of 6 experimental groups (*N* = 6 mice per group). Mice were entrained to 14:10 h light:dark cycles for 2 weeks prior to seizure testing. Zeitgeber time (ZT) 1 is defined as 1 h following lights on in the 14:10 light:dark cycle. Bmal KO mice were tested under two conditions, ZT1 and ZT15 (*N* = 7 and 6 per group, respectively).

### Seizure tests

Seizure testing was initiated when mice reached the age of 8 weeks and due to the variable effect of estrous cycle on seizure susceptibility, only male mice were studied. The electrical thresholds for generalized and maximal seizures were measured as described previously (Ferraro et al., 2011). Mice were tested with a single electric shock delivered via ear clip electrodes once per day. Sham mice received the same handling, but did not receive the shock. We used a constant current electroshock unit (model No. 7801, Ugo Basile, Varese, Italy) in which the initial current level was set at 20 mA and increased by 2 mA with each successive daily trial until a maximal seizure was observed. Other parameters of the stimulus were held constant (60 Hz, 0.4 ms pulse width, 0.2 s duration). Seizures were elicited at all current intensities utilized; lower intensities produced facial and forelimb clonus, whereas higher intensities produced generalized and maximal seizures. A generalized seizure was defined by loss of upright posture (i.e., falling over) and bilateral limb clonus. The current value at which mice first exhibited a generalized seizure was taken as the generalized electroshock seizure threshold (GEST). A maximal seizure was defined by bilateral tonic extension of hind limbs. The current value at which mice exhibited tonic hind limb extension was taken as the MEST. The sequence of responses that characterized a trial in which a maximal seizure was observed is as follows: bilateral tonic forelimb flexion, bilateral tonic hind limb flexion and bilateral tonic hind limb extension. These signs were sometimes preceded or accompanied by wild running in the observation chamber. Mice were euthanatized by cervical dislocation under CO2 anesthesia immediately after a trial in which a maximal seizure was elicited.

### Western blotting

For tissue punches of hippocampus, hemisectioned brains were embedded rostral side down in OCT media and sectioned on a Leica (CM3050) cryostat at −25°C, and a 500–1000 μm 1 mm diameter punch from Interaural 1.10 to 2.10 mm (according to Paxinos & Franklin Mouse Brain Atlas, 2nd ed.) was taken for each MEST and Sham mouse from ZT1 and ZT15. Individual hippocampal punches were homogenized in 100 μl Tris-HCl buffer with Protease Inhibitor Cocktail (Sigma P2714) for 45 s and centrifuged at 14,000 rpm for 1 min, and 5 μl lysates were loaded with 5 μl 2X loading buffer and separated on 10% Tric-HCl 0.75 mm gels and transferred to nitrocellulose membranes (Invitrogen). The membranes were blocked with 5% nonfat dry milk (Nestlé) and blotted with BMAL (Arntl) antibody (1:1000; Bethyl Laboratories) and beta-actin antibody (1:1000; Cell Signaling). Goat anti-Rabbit IR800 (1:5000; LI-COR) secondary antibody was used to visualize on Odyssey scanner (LI-COR). Intensities of protein were determined using ImageJ software (NIH).

### Data analysis

Thresholds for generalized and maximal seizures were determined as arithmetic mean values for each experimental group. ANOVA was used to examine the effect of time of day on seizure thresholds and a 2-Way ANOVA was used to include the effect of strain. *Post hoc* analyses to examine statistical relationships for seizure threshold values between individual groups were conducted using the Tukey test. For analysis of Bmal KO data, comparisons were made to values from B6 mice collapsed across lights-on and lights-off phases since there were no significant differences between groups within each phase.

Kaplan-Meier analysis for seizure endpoints was examined using MedCalc statistical software (http://www.medcalc.org), and trial time was defined as the day animals reached either generalized or maximal seizure endpoint.

For diurnal western blot studies statistical significance was determined using a two-tailed Student's *t*-test using Excel (Microsoft).

## Results

**Figure 1:**
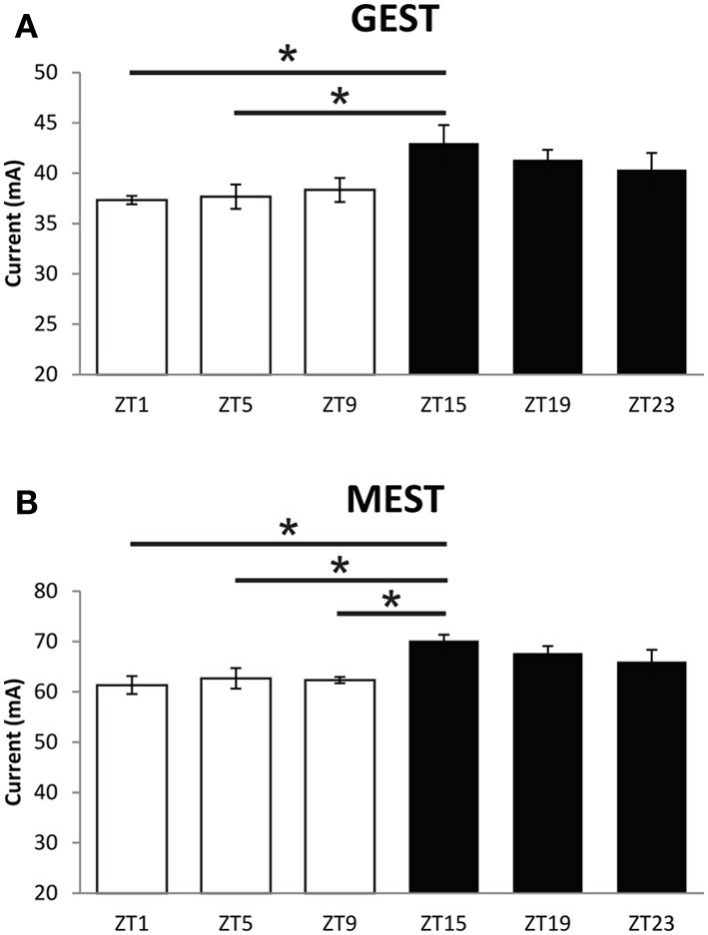
**Diurnal effects on seizure threshold**. Time-of-day variation in **(A)** generalized (GEST) and **(B)** maximal (MEST) electroshock seizure threshold. Male C57BL/6J mice (8–9 weeks of age) were housed individually and tested for electroshock seizure thresholds at different times during the 14:10 Lights-on:Lights-off diurnal cycle. One-Way ANOVA for GEST, *p* = 0.023, MEST, *p* = 0.004; (*N* = 6). Student-Newman-Keuls *post-hoc* test for all pairwise comparisons are shown for differences between groups for GEST and MEST (^*^p < 0.05). ZT, zeitgeber time. Light bars = Lights on, Dark bars = Lights off.

**Figure 2:**
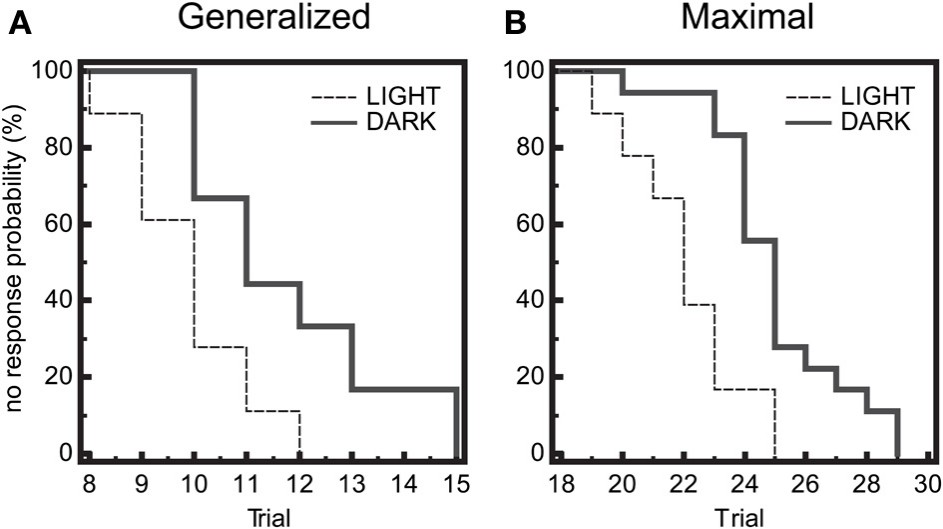
**Diurnal effects on seizure threshold**. Endpoints of electroshock-induced seizures for the three light-period groups (ZT1, 5, 9) and dark-period groups (ZT15, 19, 23) show differential percent probability of no response in both **(A)** generalized (GEST) and **(B)** maximal (MEST) electroshock seizure threshold, with greater resistance in the dark-phase (solid line) compared to the light phase (dashed line). **(A)** Median trial number to reach GEST: Light = 10, Dark = 11; Logrank test: Chi-square 11.31, *P* = 0.0008; *N* = 18 per group. **(B)** Median trial number to reach MEST: Light = 22, Dark = 25; Logrank test: Chi-square 13.96, *P* = 0.0002; *N* = 18 per group.

Seizure threshold was examined at 6 timepoints across a 14:10 light:dark cycle for diurnal fluctuations in wild-type (WT) mice. We observed a significant time-of-day dependent change in seizure threshold in both generalized (GEST) and maximal (MEST) seizures (*p* = 0.023 and *p* = 0.004, respectively, One-Way ANOVA), with elevated threshold levels in the dark phase (Figures 1A,B). Peak GEST and MEST each occurred 1 h into the dark phase at ZT15 (*p* < 0.05, Student-Newman-Keuls *post-hoc* test). Since GEST and MEST values appeared lower during the light phase compared to the dark phase overall, we were interested in testing for the diurnal effects on seizure endpoints between these two groups. We observed a significant decrease in no response probability for both GEST and MEST during the light phase compared to the dark phase (Figures 2A,B; *p* = 0.0008 and *p* = 0.0002, respectively, Kaplan-Meier analysis Logrank test).

**Figure 3:**
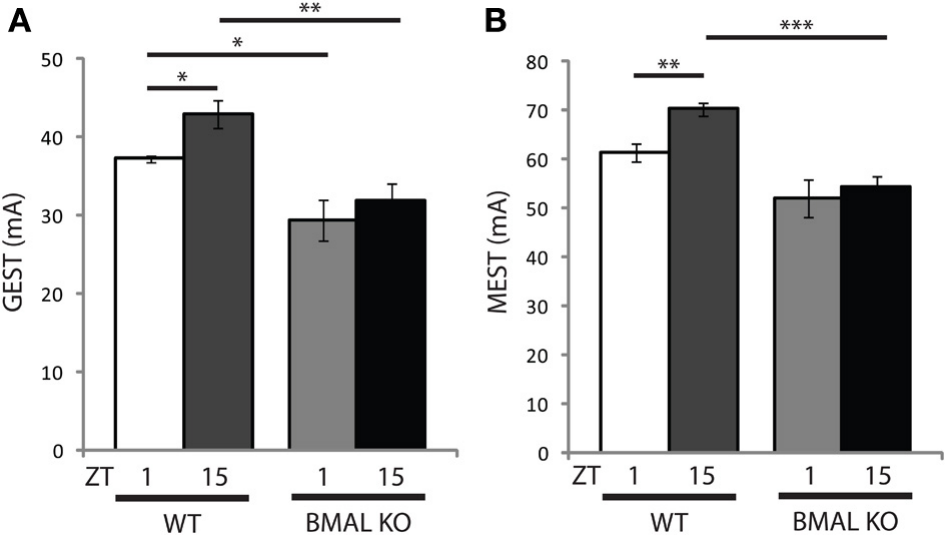
**Diurnal seizure susceptibility and baseline setpoint depends on BMAL1 expression**. Time-of-day variation in **(A)** generalized (GEST) and **(B)** maximal (MEST) electroshock seizure threshold observed in wild-type (WT) mice is blocked in BMAL KO mice. **(A)** *t*-test (two-tail) WT GEST: ZT1 vs. ZT15 ^*^p < 0.05; WT vs. BMAL KO GEST: ZT1 vs. ZT1 ^*^p < 0.05, ZT15 vs. ZT15 ^**^p < 0.01. **(B)** *t*-test (two-tail) WT MEST: ZT1 vs. ZT15 ^**^p < 0.01; WT vs. BMAL KO GEST: ZT15 vs. ZT15 ^***^p < 0.001. Error bars = s.e.m. ZT, zeitgeber time.

Circadian clock genes, such as BMAL1, are known to regulate circadian rhythms of locomotor wheel-running behavior (Bunger et al., 2000), as well as the diurnal control of sleep-wake states (Laposky et al., 2005) and memory formation (Kondratova et al., 2010), suggesting that these genes may also participate in basic neurophysiological function. Therefore we examined the effects of diurnal seizure susceptibility in BMAL1 KO mice, and found that the time-of-day variation in both GEST and MEST observed in WT mice was blocked (Figures 3A,B). Further, the baseline setpoint for seizure threshold was also significantly reduced in BMAL1 KO mice compared to WT mice for both GEST and MEST (Figures 3A,B). BMAL1 KO mice had a significant reduction in GEST irrespective of time-of-day (Figure 3A, *p* < 0.05 ZT1; *p* < 0.01 ZT15, two-tailed *t*-test), while MEST was significantly lower at ZT15 compared to WT (Figure 3B, *p* < 0.001 two-tailed *t*-test).

**Figure 4:**
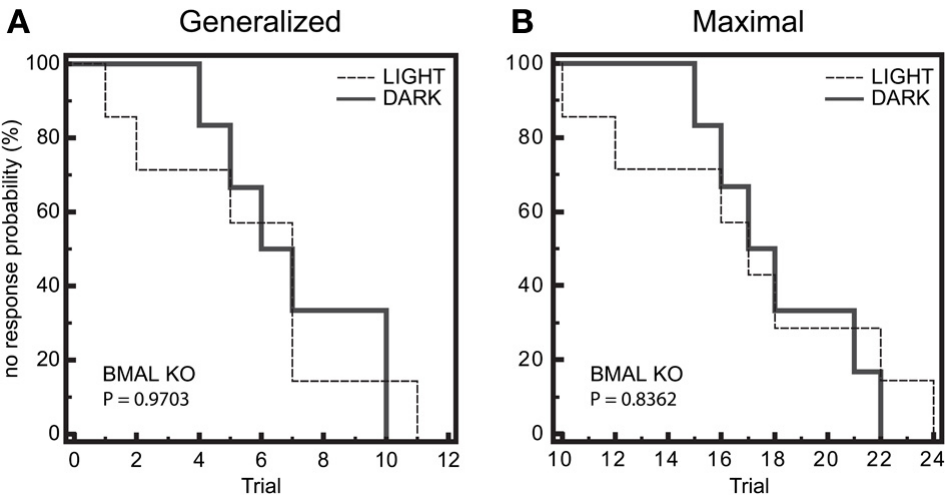
**Diurnal effects on seizure endpoints are blocked in BMAL1 KO mice. (A)** and **(B)** Endpoints of electroshock-induced seizures for the BMAL KO mice in the light-period (ZT1) and dark-period (ZT15) fail to show differential percent probability of no response in either **(A)** generalized (GEST) or **(B)** maximal (MEST) electroshock seizure threshold. **(A)** Median trial number to reach GEST: Light = 7, Dark = 7; Logrank test, *P* = 0.9703; *N* = 6–7 per group. **(B)** Median trial number to reach MEST: Light = 17, Dark = 18; Logrank test, *P* = 0.8362; *N* = 6–7 per group.

**Figure 5:**
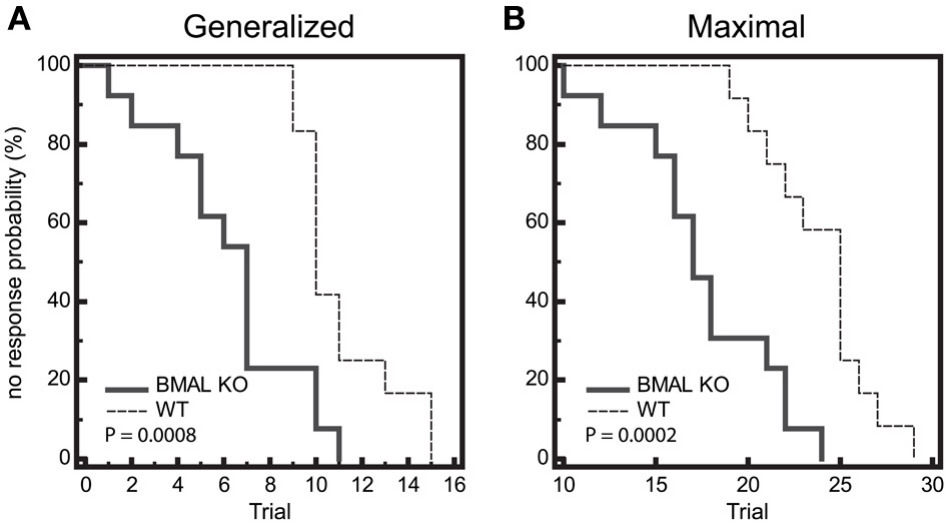
**Seizure endpoints are reduced in BMAL1 KO mice compared to WT. (A)** and **(B)** Endpoints of electroshock-induced seizures comparing BMAL KO mice and WT mice from data combined in for the light-period and dark-period (ZT1 and ZT15) show differential percent probability of no response in both **(A)** generalized (GEST) and **(B)** maximal (MEST) electroshock seizure threshold. **(A)** Median trial number to reach GEST: WT = 10, BMAL KO = 7; Logrank test, *P* = 0.0008; *N* = 12–13 per group. **(B)** Median trial number to reach MEST: WT = 25, BMAL KO = 18; Logrank test, *P* = 0.0002; *N* = 12–13 per group.

**Figure 6:**
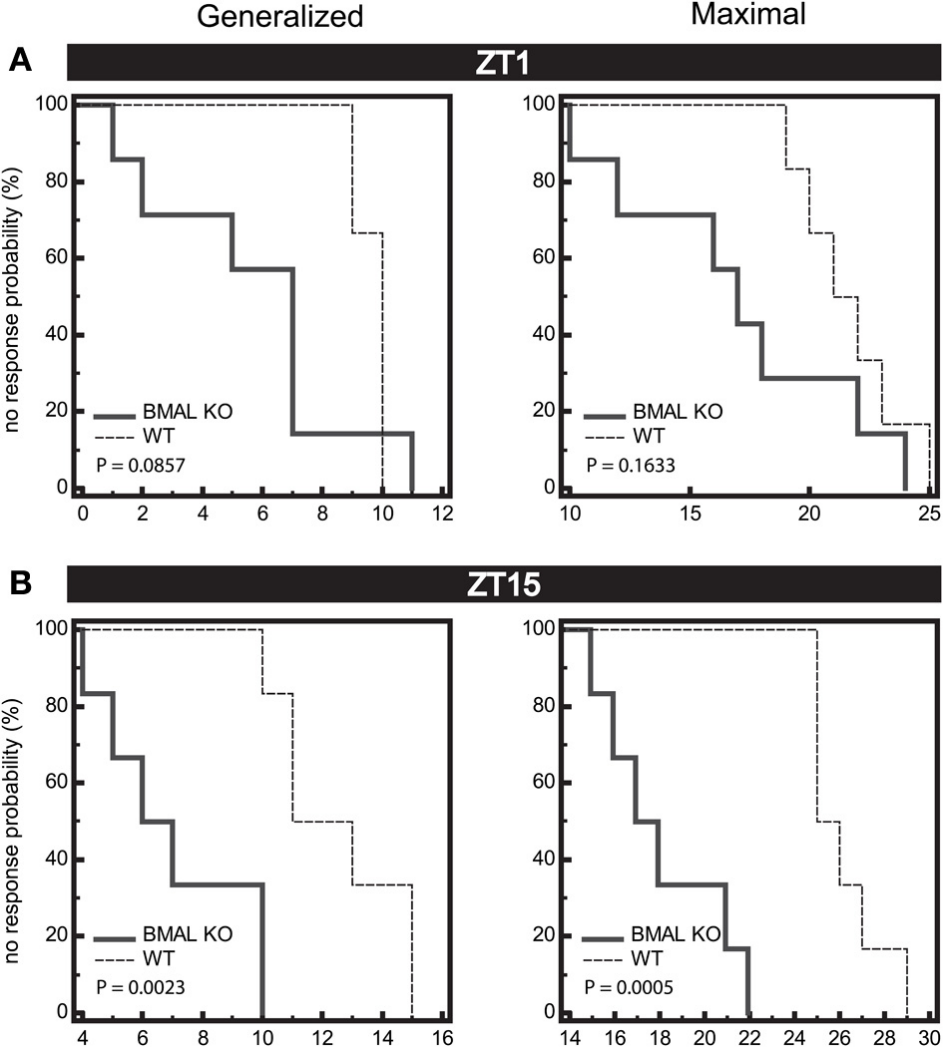
**Seizure endpoints are reduced in BMAL1 KO mice compared to WT. (A)** and **(B)** Endpoints of electroshock-induced seizures comparing BMAL KO mice and WT mice from data combined in for the light-period (**A**, ZT1) and dark-period (**B**, ZT15) show differential percent probability of no response in both generalized (GEST) and maximal (MEST) electroshock seizure threshold. **(A)** ZT1, Median trial number to reach GEST: WT = 10, BMAL KO = 7; Logrank test, *P* = 0.0857; Median trial number to reach MEST: WT = 22, BMAL KO = 17; Logrank test, *P* = 0.1633, *N* = 6 per group. **(B)** ZT15, Median trial number to reach GEST: WT = 13, BMAL KO = 7; Logrank test, *P* = 0.0023; Median trial number to reach MEST: WT = 26, BMAL KO = 18; Logrank test, *P* = 0.0005; *N* = 6–7 per group.

We next wanted to determine whether there was a difference in seizure end-point probability in BMAL1 KO mice. Given that the diurnal profile of seizure threshold is blocked in BMAL1 KO mice, we hypothesized that the diurnal variation in no response probability should also be blocked. Kaplan-Meier analysis of seizure endpoints revealed that BMAL1 KO mice do not display a difference in seizure endpoint between light and dark phase groups for either GEST or MEST (Figure 4, *p* = 0.9703 and *p* = 0.8362, respectively, Logrank test). Since BMAL1 KO mice have a reduced seizure setpoint, we hypothesized that BMAL1 KO mice should also have reduced overall seizure endpoint values compared to WT mice. Kaplan-Meier analysis of seizure endpoints comparing BMAL1 KO mice with WT mice show a significant difference in seizure endpoint for both GEST and MEST (Figure 5, *p* = 0.0008 and *p* = 0.0002, respectively, Logrank test). These strain differences on GEST and MEST were also time-of-day dependent, since seizure endpoints at ZT1 did not significantly differ between BMAL1 KO mice and WT mice, but were significant at ZT15 (Figure 6, *p* = 0.0023 and *p* = 0.0005, respectively, Kaplan-Meier analysis Logrank test).

**Figure 7:**
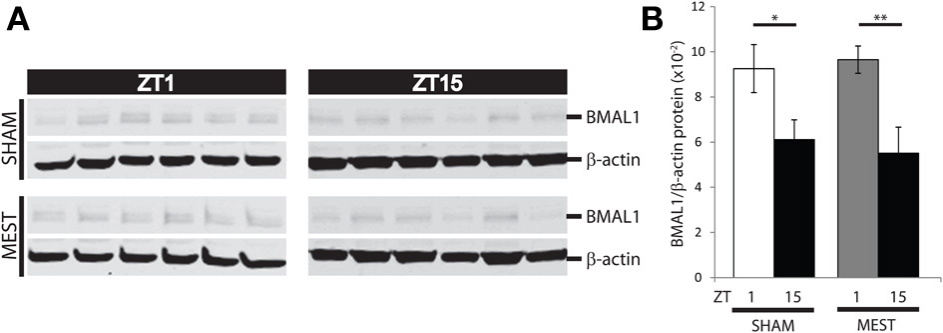
**Diurnal hippocampal BMAL1 expression is not affected by MEST**. Time-of-day variation in BMAL1 hippocampal protein expression is unaffected by MEST. **(A)** Western blot of WT mouse hippocampal BMAL1 and b-actin in SHAM controls vs. MEST at ZT1 and ZT15. **(B)** Quantification of BMAL/b-actin protein expression from western blots in **(A)**; *t*-test (two-tail) SHAM: ZT1 vs. ZT15 ^*^p < 0.05; MEST: ZT1 vs. ZT15 ^**^p < 0.01. *N* = 6 per group. Error bars = s.e.m. ZT, zeitgeber time.

Lastly, we wanted to examine whether seizure activity in turn affects time-of-day dependent BMAL1 expression in WT mice. Brains of WT mice subjected to MEST or handled controls (Sham) from ZT1 and ZT15 were dissected, and punches of hippocampi from hemisections were lysed for analysis of protein expression by western blotting. The time-of-day variation in hippocampal BMAL1 protein expression in Sham controls (Figure 7, *p* < 0.05, *t*-test) also remained following MEST (Figure 7, *p* < 0.01, *t*-test), indicating seizure activity does not influence diurnal BMAL1 expression.

## Discussion

In this study, we observed a time-of-day dependent effect on seizure threshold for both generalized and maximal seizures, with a peak in threshold at the beginning of the dark cycle. We also observed a difference in seizure endpoints between the light period and dark period, suggesting circadian factors may contribute to neural excitability. Therefore, we next examined the time-of-day effect on seizure threshold in the circadian clock mutant BMAL1 KO mice, and observed the diurnal profile of GEST and MEST was blocked. Further, we show overall seizure threshold is significantly lower in BMAL1 KO mice compared to WT mice, suggesting BMAL1 regulates baseline levels of excitability in addition to time-of-day dependent changes. Seizure endpoints for both GEST and MEST were significantly reduced in BMAL1 KO mice compared to WT mice. The diurnal hippocampal expression of BMAL1 protein was not affected by seizure activity. These findings suggest time-of-day regulates seizure susceptibility, and this effect is mediated, at least in part, by core circadian factors such as BMAL1. To the best of our knowledge, this is the first report to characterize an association between a core clock gene product and epileptogenesis.

Sleep-wake behavior and circadian rhythms are known to regulate propensity of seizures, while their relative influence likely depends on origin and seizure type (Matos et al., 2010a; Cho, 2012). For example, adults with complex partial and temporal seizures were observed to occur more frequently during the 11:00–17:00 h time period, while parietal seizures were more prevalent during the 17:00–23:00 period (Hofstra et al., 2009a). Age-specific and time-of-day dependent differences in extra-temporal seizures were observed between children and adults (Hofstra et al., 2009b), suggesting developmental regulation of the diurnal susceptibility for certain seizures. Generalized seizures in children were observed more often during sleep for tonic or tonic-clonic types, whereas absence, atonic, myoclonic, and clonic seizures occurred more readily during wakefulness (Zarowski et al., 2011). This evidence indicates that seizure type, severity, and developmental profile are all additional factors which may differentially contribute to the time-of-day variation in seizure susceptibility. Future work using animal models to examine molecular mechanisms that differentially regulate diurnal profiles between seizure subtypes may help determine better treatments tailored to individuals.

Previous studies examining the deletion of three genes that are regulated by the circadian clock show increased susceptibility to generalized spontaneous seizures and audiogenic epilepsies (Gachon et al., 2004), suggesting clock output can modulate time-of-day expression of seizures. Animal models of epilepsy also exhibit circadian rhythms in seizures (Hellier and Dudek, 1999; Fenoglio-Simeone et al., 2009; Tchekalarova et al., 2010; Matzen et al., 2012). KCNA1 KO mice show robust oscillations in diurnal seizures, with heightened levels near the dark-light transition, and a lower propensity at the light-dark transition (Fenoglio-Simeone et al., 2009), a time-of-day effect corresponding to our observation of elevations in WT mice seizure threshold in this study. Here, we observed that this diurnal influence of seizure threshold was absent, and the overall threshold was further reduced in BMAL1 KO mice. BMAL1 KO mice have disrupted rest-activity cycles under light-dark conditions (Laposky et al., 2005). Since KCNA1 KO mice also have disrupted rest-activity cycles (Fenoglio-Simeone et al., 2009), but unlike BMAL1 KO mice, have robust diurnal rhythms of seizures, these studies may point toward potential mechanisms dissociating circadian effects from sleep/wake effects. Our work demonstrates that overall seizure susceptibility is regulated by time-of-day, and this effect is mediated by a core clock gene. Future studies examining development of the diurnal profile, and relative impact of factors that specifically affect either sleep or the circadian clock will be important to disentangle the principal effect of BMAL1 on seizure threshold.

Animal models also show a reciprocal interaction between sleep and epilepsy (van Luijtelaar and Bikbaev, 2007; Matos et al., 2010a,b; Yi et al., 2012), evidence for the importance of the utility of animal models in translational research studies in this area (Matos et al., 2011). Some studies on models of limbic epilepsy have paralleled human studies in that spontaneously occurring seizures are more frequent during the light phase of the diurnal cycle. For example, pilocarpine-treated rats exhibited twice as many spontaneous seizures during the light phase as compared to the dark phase, a rhythm that remained intact and governed by the clock even when animals were maintained in constant darkness (Quigg, 2000). However, a more recent study reported spontaneous seizures elicited by pilocarpine treatment in rats was independent of the circadian cycle (Bajorat et al., 2011), an effect likely due to dependence on underlying sleep architecture (Matos et al., 2010b). In a model of electrically-induced status epilepticus, dentate gyrus neurons exhibited enhanced electrical excitability that corresponded with the time of day of enhanced seizure susceptibility (Matzen et al., 2012). Further, in a study of WAG/Rij spontaneously epileptic rats, an 8-h shift in onset of the light phase resulted in prolonged aggravation of epileptic activity, observed mostly during the light phase (Smyk et al., 2012). In a study that observed a time-of-day distribution of spontaneous motor seizures in rats, a difference in the relative number of seizures when rats were “active” vs. “inactive” also occurred independent of time-of-day (Hellier and Dudek, 1999). This indicates there may be distinct sleep/wake state behavioral components separate from circadian influence on seizure susceptibility. These studies also raise the importance of using animal models for examining the relationship between time-of-day behaviors and predisposition to seizure.

Diurnal factors, such as sleep and circadian behavior are known to impact the propensity of seizures. Many studies have reported seizure activity is increased by sleep, or is more pronounced after arousal from sleep or following sleep deprivation. Time-of-day dependent differences in seizure could result from distinct mechanisms related to either sleep or circadian factors. In addition, whether an association between core circadian clock factors and epilepsy exists is unknown. Here, we observed that seizure susceptibility and endpoints are driven strongly by time-of-day dependent factors. As a first step in elucidating potential mechanisms responsible for these observations, we identified the core-clock transcription factor BMAL1 gene to be necessary for these time-of-day differences in seizure threshold and endpoint. Further, while seizure activity itself does not influence hippocampal BMAL1 expression, we show the BMAL1 gene regulates baseline threshold for both generalized and maximal seizures. These studies implicate circadian-clock signaling pathways in mediating seizure susceptibility and neural excitability, providing needed insight into molecular mechanisms contributing to epileptogenesis (Di Maio, 2014) and a new model organism from which to study the relationship between time-of-day dependent behaviors and seizure.

### Conflict of interest statement

The authors declare that the research was conducted in the absence of any commercial or financial relationships that could be construed as a potential conflict of interest.
